# Association Between Wrist-Worn Device-Estimated Sleep Efficiency and Hand Grip Strength in Older Outpatients With Cardiometabolic Diseases: Cross-Sectional Study

**DOI:** 10.2196/95303

**Published:** 2026-10-09

**Authors:** Yoshiaki Tamura, Rui Gong, Yuji Murao, Fumino Kobayashi, Satomi Kobayashi, Remi Kodera, Kazuhito Oba, Maki Shirobe, Kenji Toyoshima, Kanae Kubo, Yuko Chiba, Hisashi Kawai, Joji Ishikawa, Hiroyuki Sasai, Yoshinori Fujiwara, Shunei Kyo, Kenji Toba, Masahiro Akishita, Shuichi Obuchi, Atsushi Araki

**Affiliations:** 1 Department of Diabetes, Metabolism, and Endocrinology, Tokyo Metropolitan Institute for Geriatrics and Gerontology, 35-2 Sakaecho, Itabashi-ku, Tokyo, 173-0015, Japan, 81 3-3964-1141, 81 3-3964-1392; 2 Center for Comprehensive Care and Research for Prefrailty, Tokyo Metropolitan Institute for Geriatrics and Gerontology, Itabashi-ku, Tokyo, Japan; 3 Research Team for Human Care, Tokyo Metropolitan Institute for Geriatrics and Gerontology, Itabashi-ku, Tokyo, Japan; 4 Department of Medicine and Rheumatology, Tokyo Metropolitan Institute for Geriatrics and Gerontology, Itabashi-ku, Tokyo, Japan; 5 Research Team for Promoting Independence and Mental Health, Tokyo Metropolitan Institute for Geriatrics and Gerontology, Itabashi-ku, Tokyo, Japan; 6 Department of Cardiology, Tokyo Metropolitan Institute for Geriatrics and Gerontology, Itabashi-ku, Tokyo, Japan; 7 Tokyo Metropolitan Institute for Geriatrics and Gerontology, Itabashi-ku, Tokyo, Japan

**Keywords:** older, wearable devices, wrist-worn, sleep efficiency, grip strength, diabetes mellitus

## Abstract

**Background:**

Older outpatients with chronic diseases are susceptible to muscle weakness and functional disabilities. Recently, various wearable devices have been developed to generate health lifelogs in daily life; however, data from patients with chronic diseases remain scarce. Previous studies have shown that subjective sleep efficiency assessed by self-questionnaires is associated with grip strength, but whether objective sleep efficiency detected by wearable devices is associated with physical function and body composition remains unclear.

**Objective:**

The aim of this study is to clarify the association between sleep duration, sleep efficiency estimated by wearable watch, grip strength, frailty, and sarcopenia in outpatients with predominantly cardiometabolic diseases and the influence of diabetes as part of the Smart Watch Innovation for Geriatrics and Gerontology (SWING-Japan) project.

**Methods:**

In this cross-sectional study, the wrist-worn device Silmee 22 was used in an outpatient clinic of an acute care facility for 7‐90 nights for the analyses, and individual average sleep duration and efficiency (wearable-estimated sleep efficiency: total sleep duration/time spent in bed) were calculated. Low grip strength was defined as <28 kg for men and <18 kg for women. Sarcopenia was defined based on the Asian criteria and frailty based on the Japanese version of Cardiovascular Health Study and Kihon Checklist criteria. Sleep indices were compared between individuals with and without these conditions. Next, logistic regression analysis was performed, with lower grip strength as the dependent variable and wearable-estimated sleep efficiency as the independent variable, adjusted for age, sex, BMI, glycohemoglobin, cognitive function (Mini-Mental State Examination), Geriatric Depression Scale-15 score, and daily step count measured using the ankle-worn device WalkX. Subgroup analyses were performed and stratified according to the status of diabetes mellitus.

**Results:**

Data from 165 patients (median age: 77 y; duration of device use: 65 d; diabetes prevalence: 55%) were used for analyses. Wearable-estimated sleep efficiency and sleep duration were significantly lower in patients with low grip strength than in those without (58.0% vs 62.7%, *P*=.006, 245 vs 276 min, *P*=.04). Wearable-estimated sleep efficiency was not associated with frailty, low muscle mass, or slow walking speed. After adjusting for the above variables, low wearable-estimated sleep efficiency, but not low sleep duration, was associated with a risk of lower grip strength (odds ratio 1.04, 95% CI 1.01‐1.08; *P*=.02) by 1 percentage point decrease in wearable-estimated sleep efficiency. This association was observed in patients with diabetes (odds ratio 1.05, 95% CI 1.002‐1.093; *P*=.04). However, no significant interaction was observed.

**Conclusions:**

In older patients with cardiometabolic diseases, objective low wearable-estimated sleep efficiency evaluated with wrist-worn wearable devices was associated with low muscle strength. No evidence was found to suggest that the strength of this association differed according to diabetes status. Longitudinal studies are needed to clarify the long-term association between low wearable-estimated sleep efficiency and muscle strength in this population.

## Introduction

Sarcopenia is a state defined as muscle weakness and muscle loss. Between these, it has been shown that low muscle strength is the strongest prognostic factor. In the Health ABC Study, Newman et al [1] showed that grip strength and knee extension strength were associated with mortality, whereas thigh muscle area measured by computed tomography was not. It is estimated that older outpatients with chronic diseases are more susceptible to sarcopenia. To date, various clinical parameters that can be measured in outpatient clinics have been associated with low muscle strength. For example, in a Japanese community cohort-based study, that included bone area ratio, forced expiratory volume, estimated glomerular filtration rate, and diastolic blood pressure [2]. However, the values at medical centers or outpatient clinics are measured under specific conditions and may not necessarily represent those in daily activities.

Recently, wearable devices worn on various parts of the body, including the wrist, ankle, finger, back, and thigh, have been developed to continuously and accurately monitor various users’ health lifelogs, such as pulse rate, body temperature, physical activity, and walking speed in their daily living [3]. Moreover, some devices can evaluate users’ sleep patterns that cannot be assessed in outpatient clinics. Recently, we established the Smart Watch Innovation for Next Geriatrics and Gerontology (SWING-Japan) study to investigate the association between various health lifelogs extracted from wrist-worn devices and frailty, sarcopenia, and related factors among community-dwelling older adults and outpatients. As a part of this project, we present our findings on the association between sleep indices detected by the devices and the above outcomes.

Conventionally, sleep efficiency is evaluated using a self-questionnaire, and some studies have shown an association between subjective sleep efficiency and physical functions such as grip strength [4,5]. Another report has shown that subjective sleep efficiency is associated with grip strength in patients with diabetes, but the sleep indices in these studies were not objectively evaluated [6]. More recently, some reports have shown that objective sleep state indices, which can be evaluated using devices such as polysomnography [7] or actigraphy [8], are also associated with frailty. However, these devices are difficult to handle in daily life and using them and extracting data from them require special techniques or software [9]. Today, many low-cost wearable devices have been developed. Compared to actigraphs, they are easy to wear and able to monitor health lifelogs in real-life settings over a long duration across many outpatients. Some of them have been shown to have fair reliability of sleep or activity data. In this cross-sectional study, we evaluated the association between objective sleep status as measured by consumer-grade and easy-to-wear wearable devices and grip strength in outpatients of the Frailty Clinic and the influence of diabetes, as well as the association between sleep indices and frailty and component factors of sarcopenia.

## Methods

### Participants

Older outpatients who regularly visited the Tokyo Metropolitan Institute for Geriatrics and Gerontology for the treatment of chronic diseases, mainly cardiometabolic diseases (such as diabetes, hypertension, and dyslipidemia), participated in this study. Our institution has a Frailty Clinic, where outpatients and inpatients who are suspected of or worried about frailty can be evaluated for their physical and cognitive functions [10]. We made a brochure for this study, and those who were interested were introduced to the Frailty Clinic. Patients were registered between December 2022 and May 2024.

The inclusion criteria were patients aged ≥60 years who agreed to setting up a gateway in their home to upload the health lifelog data to the cloud. The exclusion criteria were as follows: patients with severe psychiatric diseases that may interfere with the stable wearing of devices; those with vascular shunts for hemodialysis in the forearm or postsurgical state of breast cancer; those with severe cognitive impairment; and those deemed inappropriate for participation.

### Wearable Devices

This analysis was conducted as part of the SWING-Japan study, an intensive investigation examining the association of behavioral data, such as physical activity and sleep patterns monitored by wearable devices, with physical and mental health outcomes among older adults [11]. We used Silmee W22 (TDK Corporation), a wrist-worn watch-type device, to detect sleep status [12]. A validation study for the accuracy of the device in evaluating sleep status, comparing the data between the device and a portable electroencephalography (EEG), was recently performed by a group at our institute [12]. They concluded that when averaging the measurements over nights, the agreement between total sleep time, wake after sleep onset, and sleep efficiency improved to the level of “fair to good,” with intraclass correlation coefficients of 0.75, 0.47, and 0.49, respectively. However, as it is not yet equivalent to those in EEG-derived sleep efficiency, we defined sleep efficiency derived from Silmee data as “wearable-estimated sleep efficiency” in this report. To evaluate step count, an ankle-band device, WalkX (ACOS Co Ltd), was simultaneously worn on top of the participant’s lateral malleolus. WalkX has a 3D accelerometer, and another group at our institute recently validated the accuracy of the walking speed measured by this device [13].

At the start of the study, the technical assistant fully instructed the patients on how to use the devices and displayed the application video. The patients were instructed to wear the devices as long as possible, except during bathing (for charging). Most patients had their own smartphones and could directly check their health lifelogs, including walking state and pulse. A monthly report showing the summary of their health lifelog was sent to patients who did not have smartphones. This study was conducted using baseline data from a 1-year longitudinal study, and patients were instructed to wear the devices for at least 3 months.

### Definition of Valid Data and Sleep State

We excluded whole-day data for Silmee and WalkX if the wearing time was less than 12 hours. We defined the going-to-bed time as the earliest time point after 18:00 at which the status on the smartwatch turned from “awake” to “sleep” and this state was sustained for at least 30 consecutive minutes. Likewise, we defined the wake-up time as the latest time point before 9:00 when the status on the smartwatch turned from “sleep” to “awake” and this state was sustained for at least 30 consecutive minutes. The sleeping time was defined as the duration between going to bed and waking. Wearable-estimated sleep efficiency (%) was defined as the total sleep duration divided by the amount of time spent in bed (ie, the duration between going to bed and waking up).

Naps were excluded from sleep duration and wearable-estimated sleep efficiency according to the wake-sleep transition criteria described previously. Periods of device nonwear and charging were distinguishable from sleep. Therefore, if no sleep data were recorded after 18:00 because of device nonwear or charging, the day was treated as having missing sleep data. If a participant woke up after 9:00, the wake-up time was considered missing. However, we confirmed that more than 90% of participants woke before 9:00 and went to bed after 18:00. Nighttime awakenings were considered wake periods while in bed.

For both sleep and step data, the average values of the valid days were used as individual data for the analysis.

### Indicators of Rest–Activity Rhythm

Nonparametric circadian rhythm variables were calculated from Silmee data according to the method described by van Someren et al [14]. Interdaily stability (IS) and intradaily variability (IV) indicate synchronization of the circadian timing system and fragmentation of the rest-activity rhythm, respectively. M10 was defined as the mean activity level during the consecutive 10-hour period with the highest physical activity, whereas L5 was defined as the mean activity level during the consecutive 5-hour period with the lowest activity. Relative amplitude (RA), an indicator of the contrast between daytime and nighttime activity, was calculated as RA = (M10 - L5)/(M10+ L5). The averages of these variables over 7 consecutive days beginning on the second day of device wear (IS_7, IV_7, M10_7, L5_7, and RA_7) were used for the analyses.

### Sarcopenia, Physical Function, and Frailty

Grip strength was measured using a dynamometer (TAKEI Scientific Instruments, SANKA). The participants completed two tests with both hands, and the highest value was recorded. Muscle mass was evaluated by bioimpedance analysis using InBody 770 (INBODY Japan). The appendicular skeletal muscle index (SMI) was calculated as the total appendicular muscle mass (kg) divided by the square of the height (m). Walking speed was calculated as the time spent during a 4-meter walk at the usual speed, allowing 1 meter for acceleration and 1 meter for deceleration. The participants took the walking test twice, and the highest value was recorded. Sarcopenia was defined according to the diagnostic criteria of the Asian Working Group for Sarcopenia (AWGS 2019), namely low SMI and either low grip strength or low walking speed [15]. A low grip strength was defined as <28 kg in men and <18 kg in women, and a low SMI as <7.0 kg/m^2^ in men and <5.7 kg/m^2^ in women, respectively, both of which are key criterion for the diagnosis of sarcopenia. Similarly, low walking speed was defined as <1.0 m/s.

Frailty was diagnosed according to the Japanese version of the Cardiovascular Health Study (J-CHS) [16] and Kihon Checklist (KCL) criteria [17]. The J-CHS comprises 5 items: unintentional weight loss, low grip strength, slow walking speed, low activity, and fatigue. Those patients who present with 3 or more items are identified as frail. The KCL is a multidimensional, comprehensive geriatric assessment-based assessment of frailty comprising 25 questions. Individuals who score 8 or more are identified as frail.

### Other Clinical Assessments and Blood and Urine Sampling

Information on participants’ comorbidities and medications (such as diabetes and its duration, hypertension, and dyslipidemia) was extracted from their medical records. Blood and urine samples were collected at the closest visit to the start time of device use. Cognitive function was evaluated using the Mini-Mental State Examination (MMSE), and depressive mood was assessed using the Geriatric Depression Scale (GDS)-15, as previously described [10].

### Statistics

We extracted the health lifelog data only from days that included data for more than 12 hours. Patients with less than 7 days of health lifelog data were excluded from the analyses. In addition, those with fewer than 7 night-time data points were also excluded. This threshold was defined by referring to previous reports with actigraph data that showed that at least 7 days of recording were needed to estimate the main measures of sleep [18,19]. Data from up to 90 days were used for analyses.

Numerical variables between the two groups were compared using the Mann-Whitney *U* test, and categorical variables were compared using the *χ*² test or Fisher exact test. Correlations between two continuous variables were evaluated using the Spearman rank correlation coefficient.

For multivariable analysis, logistic regression analyses were performed, with frailty, sarcopenia, and diagnostic items of these statuses as objective variables and indices of sleep (total sleep duration and wearable-estimated sleep efficiency) as explanatory variables. Several models with covariates were used: (1) Model 1 (crude model), Model 2 (adjusted for age and sex), and Model 3 (further adjusted for BMI, glycohemoglobin, MMSE scores, average daily step count measured using WalkX, and GDS-15 score). Subgroup analyses were performed by dividing the patients into groups with and without diabetes.

To evaluate the linear assumption of wearable estimated sleep efficiency, we fitted restricted cubic spline (RCS) models with 4 knots to the logistic regression, illustrating the relationship between wearable-estimated sleep efficiency and low grip strength in total and the subgroups stratified by the diabetic state, adjusting for all the covariates above. We also performed multiple linear regression analyses to investigate the association between sleep quality and grip strength as continuous variables.

To exclude the influences of including the data of patients with relatively short wearing durations, those taking sleeping medication, and those with sleep apnea syndrome (SAS), three sensitivity analyses were further performed: (1) including only the participants who wore the Silmee for ≥14 nights or ≥30 nights, (2) excluding those who take sleep medications or antipsychotics, and (3) excluding those with SAS. In addition, among participants with diabetes, logistic regression analyses were repeated after further adjusting Model 3 for diabetes duration and the use of glucagon-like peptide-1 (GLP-1) receptor agonists or sodium-glucose cotransporter 2 (SGLT2) inhibitors, which may affect muscle mass. We also performed a sensitivity analysis by additionally including nonparametric circadian rhythm indices (IV_7, IS_7, M10_7, L5_7 and RA_7) in Model 3 for the overall cohort.

Statistical analyses were performed using SPSS (version 20; IBM Corp) and R software (version 4.5.1; R Foundation for Statistical Computing). *P* values were considered statistically significant at *P*<.05.

### Ethical Considerations

Written informed consent was obtained from all the participants. The study protocol was approved by the Ethics Committee of the Tokyo Metropolitan Institute for Geriatrics and Gerontology (approval number: R 22‐035). All patients’ data were anonymized to protect their personal information.

## Results

### Patient Characteristics

A flowchart of the patient selection process is shown in Figure 1. Among the 225 registered patients, those with incomplete Silmee, WalkX, or other clinical data were excluded. Among them, those with incomplete night data and those younger than 65 years of age were excluded, and the data from the remaining 165 patients were used for analysis.

Patient characteristics are shown in Table 1. The median age was 77 years old, and 42% (n=69) of the participants were men. The prevalence rates of diabetes, dyslipidemia, and hypertension were 55% (n=90), 73% (n=120), and 64% (n=105), respectively. The prevalence of J-CHS–defined frailty, KCL-defined frailty, low grip strength, low SMI, low walking speed, and sarcopenia were 6.1% (n=10), 7.9% (n=13), 24.7% (n=40), 39.4% (n=63), 13.9% (n=23), and 17.1% (n=27), respectively (Table 1). Patients who used sleep medications and antipsychotics represented 9.1% (n=15) of the study population. There were 11 patients with SAS (6.7%) and 2 patients with restless leg syndrome (1.2%), according to the medical record.

**Figure 1.**
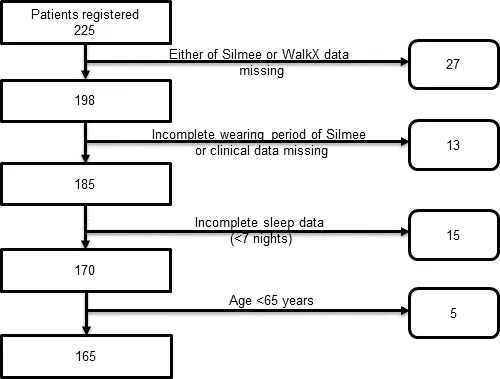
Flow chart of patient selection. Older outpatients with chronic diseases were recruited to the Frailty Clinic. Wrist-worn and ankle-band devices were worn up to 90 days. Data were cross-sectionally evaluated.

**Table 1.** Clinical characteristics of study participants (N=165).

Characteristics	Values
Age (years), median (IQR)	77 (73-82)
Women, n (%)	96 (58.2)
BMI (kg/m^2^), median (IQR)	23.4 (21.2‐25.6)
Systolic blood pressure (mm Hg), median (IQR)	135 (124-144)
Diastolic blood pressure (mm Hg), median (IQR)	74 (67-81)
Albumin (g/dL), median (IQR), n=161	4.2 (4.1‐4.4)
Glycated hemoglobin (%), median (IQR), n=152	6.6 (5.9‐7.10)
Triglyceride (mg/dL), median (IQR), n=158	116 (86-152)
LDL-C^a^ (mg/dL), median (IQR), n=156	102 (84-121)
HDL-C^b^ (mg/dL), median (IQR), n=152	60 (51-70)
eGFR-cre^c^ (mL/min/1.73m^2^), median (IQR), n=161	56.1 (48.1‐63.1)
MMSE^d^, median (IQR)	29 (28-30)
Diabetes, n (%)	90 (55)
Hypertension, n (%)	105 (64)
Dyslipidemia, n (%)	120 (73)
Frailty (J-CHS^e^), n (%)	10 (6.1)
Frailty (KCL^f^), n (%)	13 (7.9)
Grip strength (men) (kg), median (IQR), n=68	32.0 (28.7‐36.1)
Grip strength (women) (kg), median (IQR), n=94	21.2 (17.8‐23.5)
Low grip strength, n (%), n=162	40 (24.7)
SMI^g^ (men) (kg/m^2^), median (IQR), n=67	7.3 (6.8‐7.8)
SMI (women) (kg/m^2^), median (IQR), n=93	5.8 (5.4‐6.1)
Low SMI, n (%), n=160	90 (39.4)
Walk speed (m/s), median (IQR)	1.22 (1.08‐1.37)
Low walk speed, n (%)	23 (13.9)
Sarcopenia (AWGS^h^ 2019), n (%), n=158	27 (17.1)
Sleep duration (minutes), median (IQR)	437 (372-484)
Real sleep duration (minutes), median (IQR)	262 (224-332)
Wearable-estimated sleep efficiency (%), median (IQR)	60.7 (54.7‐77.0)
Average daily steps, median (IQR)	4815 (3163‐6891)
Sleep medication or antipsychotics, n (%), n=164	15 (9.1)
Sleep apnea syndrome, n (%)	11 (6.7)

a LDL-C: low-density lipoprotein cholesterol.

b HDL-C: high-density lipoprotein cholesterol.

c eGFR-cre: creatinine-based estimated glomerular filtration rate.

d MMSE: Mini-Mental State Examination.

e J-CHS: Japanese version of the Cardiovascular Health Study criteria.

f KCL: Kihon Checklist.

g SMI: skeletal mass index.

h AWGS: Asian Working Group for Sarcopenia.

### Frequency and Duration of Device Usage

Among the remaining 165 patients, the median duration of wearing Silmee was 65 days, and the median duration of wearing WalkX was 90 days (most patients wore the devices for the entire period). A histogram of valid night counts (Figure S1 in Multimedia Appendix 1) showed a bimodal curve with two peaks between 10 and 15 days and 75 and 80 days. The rates of outpatients who wore the Silmee device for ≥14 and ≥30 nights were 90% (n=149) and 77% (n=127), respectively.

### Sleep Duration and Wearable-Estimated Sleep Efficiency and Daily Steps

The median daily average duration spent in bed, total sleep duration, and wearable-estimated sleep efficiency were 437 minutes, 262 minutes, and 60.7%, respectively. The median number of daily steps was 4815 (Table 1).

### Comparisons of Wearable-Device Sleep Indices Between Physical Function Statuses

Wearable-estimated sleep efficiency was significantly lower in participants with low grip strength vs without (58% vs 62.7%; *P*=.006) (Table 2). When dividing these patients into groups by diabetic status, a difference in sleep efficiency between those with low grip strength and without was found in patients with diabetes (57.8% vs 68.1%; *P*=.006), whereas no differences were observed in those without diabetes (Figure 2). Sleep duration was also shorter in participants with low grip strength (245 vs 276 min, *P*=.04). Wearable-estimated sleep efficiency did not differ between those with and without frailty according to the J-CHS or KCL definitions; furthermore, it was not different between those with and without low walking speed, low SMI, and AWGS-defined sarcopenia. When we performed a multiple linear regression analysis with grip strength treated as a continuous variable, the direction of the association remained consistent, although it did not reach statistical significance. As there have been some reports that either extremely longer or shorter sleep duration may be a risk factor for frailty [19], we divided total sleep duration into three categories (short: <4 h, middle: 4-<7 h, and long: ≥7 h). However, we did not find a U-shaped distribution in the plots between sleep duration and risk of frailty, and we found that neither long nor short total sleep duration was associated with any of these indices of physical function (Table 3).

**Table 2.** Wearable-estimated sleep efficiency (%) between patients with and without frailty and sarcopenia and its components.

	Without the item listed (–), median (IQR)	With the item listed (+), median (IQR)	*P* value
Frailty (J-CHS^a^)	60.7 (54.7‐78.3)	59.5 (49.1‐63.1)	.14
Frailty (KCL^b^)	60.2 (54.8‐78.2)	62.7 (48.6‐76.0)	.62
Sarcopenia (AWGS^c^ 2019)	61.5 (54.5‐78.5)	59.3 (55.8‐68.3)	.46
Low grip strength	62.7 (55.8‐78.7)	58.0 (52.7‐64.0)	.006
Low SMI^d^	60.7 (53.8‐78.4)	61.5 (56.5‐75.5)	.81
Low walking speed	61.0 (55.0‐78.1)	60.2 (51.3‐68.3)	.24

a J-CHS: Japanese version of the Cardiovascular Health Study criteria.

b KCL: Kihon Checklist.

c AWGS: Asian Working Group for Sarcopenia.

d SMI: skeletal mass index.

**Figure 2.**
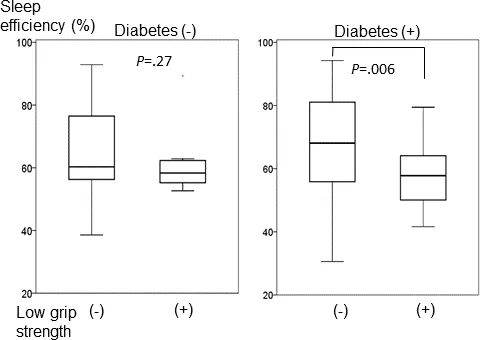
Comparison of wearable-estimated sleep efficiency between the patients with and without low grip strength stratified by the state of diabetes.

**Table 3.** Prevalence of short (S), middle (M), and long (L) sleep duration (%) between patients with and without frailty and sarcopenia and its components. Sleep duration categories were defined as follows: S: <4h, M: 4-<7h, L: ≥7h.

	Without the item listed (–), %	With the item listed (+), %	*P* value
Frailty (J-CHS^a^)	S: 37, M: 56, L: 7	S: 30, M: 70, L: 0	.87
Frailty (KCL^b^)	S: 38, M: 55, L: 7	S: 23, M: 77, L: 0	.40
Sarcopenia (AWGS^c^)	S: 34, M: 59, L: 7	S: 41, M: 52, L: 7	.71
Low grip strength	S: 34, M: 58, L: 7	S: 43, M: 53, L: 5	.70
Low SMI^d^	S: 35, M: 58, L: 7	S: 37, M: 57, L: 6	>.99
Low walking speed	S: 38, M: 56, L: 6	S: 26, M: 65, L :9	.52

a J-CHS: Japanese version of the Cardiovascular Health Study criteria.

b KCL: Kihon Checklist.

c AWGS: Asian Working Group for Sarcopenia.

d SMI: skeletal mass index.

### Multivariable Logistic Regression Analysis

In logistic regression analysis, wearable-estimated sleep efficiency was significantly associated with lower grip strength (Model 1=crude model, odds ratio [OR] 1.04, 95% CI 1.01‐1.07; *P*=.002) by 1% decrease in wearable-estimated sleep efficiency (Table 4). Significance remained after adjustment for age and sex (Model 2, OR 1.05, 95% CI 1.01‐1.08; *P*=.004), as well as after further adjustment for BMI, glycated hemoglobin, MMSE scores, daily step count, and GDS-15 (Model 3, OR 1.04, 95% CI 1.01‐1.08; *P*=.02). The significance remained even when we added hypertension, dyslipidemia, or estimated glomerular filtration rate (eGFR) to Model 3 (data not shown). No significant associations were observed between total sleep duration and outcomes, including grip strength. Wearable-estimated sleep efficiency was not associated with frailty, sarcopenia, physical function indices, or body composition. By stratifying the patients by diabetes status, we found an association between wearable-estimated sleep efficiency and muscle strength in patients with diabetes (Model 3, OR 1.05, 95% CI 1.002‐1.093; *P*=.039), but not in patients without diabetes (Table S1 in Multimedia Appendix 2). Furthermore, in patients with diabetes, the difference remained significant when we added hypertension, dyslipidemia, eGFR, urinary albumin, and the use of antidiabetic drugs to Model 3 (data not shown). However, no significant interaction was observed between participants with and without diabetes (*P* for interaction=.46; Model 3).

In RCS models with 95% CIs, nonlinearities were not significant in all 3 groups (*P*=.79, .35 and .88, respectively, for total, patients with diabetes, and patients without diabetes), indicating that the linear model is sufficiently suitable and nonlinear components did not improve model fit (Figure S2 in Multimedia Appendix 3).

**Table 4.** Binominal logistic regression analysis for the association between wearable-estimated sleep efficiency and low grip strength.

	Model 1^a^		Model 2^b^		Model 3^c^	
	Odds ratio (95% CI)	*P *value	Odds ratio (95% CI)	*P *value	Odds ratio (95% CI)	*P *value
WESE^d^,^e^	1.04 (1.01‐1.07)	.004	1.05 (1.01‐1.08)	.004	1.04 (1.01‐1.08)	.02
Age			1.08 (1.02‐1.15)	.01	1.07 (0.997‐1.150)	.06
Sex (men)			0.85 (0.39‐1.84)	.68	0.91 (0.38‐2.14)	.82
BMI					0.96 (0.85‐1.08)	.51
Glycated hemoglobin					0.82 (0.49‐1.37)	.43
MMSE^f^					1.03 (0.78‐1.37)	.81
Daily steps (per 1000 steps)					0.93 (0.77‐1.12)	.44
GDS^g^-15					0.96 (0.82‐1.14)	.65

a Model 1: crude model.

b Model 2: adjusted for age, sex.

c Model 3: adjusted for age, sex, BMI, glycated hemoglobin, MMSE, and daily steps.

d WESE: wearable-estimated sleep efficiency.

e 1-percentage-point decrease

f MMSE: Mini-Mental State Examination.

g GDS: Geriatric Depression Scale.

### Sensitivity Analyses

In the sensitivity analysis that included only the participants who wore the Silmee device for ≥14 nights or ≥30 nights, similar results were seen for the associations between wearable-estimated sleep efficiency and low grip strength when the Mann-Whitney *U* test and logistic regression analyses were carried out (Tables S2-5 in Multimedia Appendix 2). Also, in the sensitivity analyses, which excluded those outpatients who took sleep medications or antipsychotics and those with SAS, we found that these results remained unchanged (Tables S6-9 in Multimedia Appendix 2). Moreover, excluding those with restless leg syndrome did not affect the results (data not shown). In all sensitivity analyses, no significant interaction according to diabetes status was observed. Among participants with diabetes, additional adjustment for diabetes duration or the use of GLP-1 receptor agonists or SGLT2 inhibitors in Model 3 resulted in marginally significant associations between wearable-estimated sleep efficiency and low grip strength (OR 1.041, 95% CI 0.995‐1.090; *P*=.08, and OR 1.042, 95% CI 0.998‐1.089; *P*=.06, respectively).

### Influence of Rest–Activity Rhythm

Participants with low grip strength had significantly higher IV than those without low grip strength (*P*=.04). However, no significant differences were observed for the other rest-activity rhythm indices. All indices were significantly correlated with wearable-estimated sleep efficiency (IS_7: rs=0.268, *P*=.001; IV_7: rs=−0.206, *P*=.009; M10_7: rs=0.175, *P*=.03; L5_7: s=−0.190, *P*=.02; RA_7: s=0.216, *P*=.006). In the logistic regression analyses, the association between wearable-estimated sleep efficiency and low grip strength remained statistically significant regardless of which rest-activity rhythm variable was added to the model. As a representative example, the results after adding RA are shown in Table S10 in Multimedia Appendix 2.

## Discussion

### Principal Findings

In this cross-sectional study, we found that wearable-estimated sleep efficiency was significantly associated with low grip strength in older outpatients. By subgroup analyses, this association was observed in patients with diabetes; however, no significant interaction was found. From the results of RCS, this association appears to be sufficiently suitable in the linear model.

This result is consistent with the results of a systematic literature review performed by Pana et al [9], which showed that low sleep efficiency is associated with lower grip strength. However, most studies used self-administered questionnaires rather than wearable devices to evaluate sleep efficiency. Hayashi et al [6] reported an association between sleep efficiency and grip strength in inpatients with diabetes, using the Pittsburgh Sleep Quality Index (PSQI) to evaluate sleep efficiency.

In the present study, we evaluated objective sleep efficiency using a wearable device. Previous studies have reported discrepancies between objective sleep efficiency measured by actigraphy and subjective sleep quality assessed using the PSQI in older adults aged >55 years [20]. This discrepancy could be attributed to the fact that the accuracy of subjective sleep efficiency tends to be affected by patients’ memory function. There have also been reports that objective sleep efficiency is more strongly correlated with various outcomes than subjective sleep efficiency. For example, Wiranto et al [21] reported that executive function was better correlated with sleep efficiency recorded by actigraph than with total PSQI scores in men. As for physical function, a study targeting older veterans showed that objective measures of sleep recorded with an actigraph were well associated with physical function [22]. In contrast, a recent study by Shinmura et al [23] showed that subjective sleep quality using the PSQI was better associated with J-CHS–defined and KCL-defined frailty in community-dwelling older patients as compared with actigraph.

Taking these findings together, we inferred that it is recommendable that objective measures be recorded to evaluate sleep efficiency. The actigraph used in many of the studies noted above is a wrist-worn device with an acceleration sensor that can record the status of wakefulness and sleep accurately. However, using actigraphs in numerous outpatients is challenging; specific software is needed to extract data and the device is expensive because it is not generally used. Smartwatches are not expensive, and patients can easily determine their sleep status when they wake up. To date, no study has validated the accuracy of data from Silmee by directly comparing its sleep data with those of an actigraph, although data on physical activity have recently been validated [15]. Deguchi et al [12] have recently shown that sleep measures using this smartwatch showed moderate agreement when comparing its data with those from electroencephalography, provided that averaged data are used. However, as it is not equivalent, we used the term “wearable-estimated sleep efficiency” for data derived from the Silmee device. We found that the number of valid days was distributed bimodally. However, the main findings were robust to stricter thresholds in sensitivity analyses, excluding those with short wearing duration (<14 d and <30 d). This indicates that setting the minimum wearing duration to 7 days is reasonable, as has been previously shown [18,19]. Using sleep medications or antipsychotics may also influence sleep quality. However, their impact seems minimal, as another sensitivity analysis excluding patients using these medications showed the same results as those obtained from the original study population.

Nevertheless, the limited validity of the Silmee device may have influenced the results. Measurement error may have introduced regression dilution bias and nondifferential misclassification, both of which generally attenuate observed associations. Therefore, these findings should be interpreted with caution. To further address this limitation, we performed additional analyses using nonparametric circadian rhythm indices, which are well-established measures of rest-activity rhythm [24] and have been associated with functional impairment in older adults [25]. Although all rest-activity rhythm indices were significantly correlated with wearable-estimated sleep efficiency, the association between wearable-estimated sleep efficiency and low grip strength remained significant after adjustment for these variables in the multivariable analyses. These findings further support the robustness of the observed association despite the limited validity of the Silmee measurements.

We found that wearable-estimated sleep efficiency was more strongly associated with grip strength than with actual sleep duration, although it has been shown that short or long sleep duration is associated with physical dysfunction [26]. The reason for this is unclear. However, it could be that the sleep duration in many studies is self-reported and includes sleep interruption; thus, it may not reflect real sleep duration. It is likely that the association between low sleep efficiency and muscle strength is more pronounced in older adults than younger adults [27]. Several mechanisms may have influenced the association between sleep efficiency and muscle strength. Low sleep efficiency may lead to decreased secretion of anabolic hormones such as testosterone and insulin-like growth factor-1 or an increase in catabolic hormones such as cortisol. A previous study showed that the sleep quality evaluated by the PSQI is correlated with testosterone levels [28], while another found that the difference in serum cortisol levels between midnight and the morning was associated with sleep efficiency as measured by actigraphy [29]. Furthermore, sleep efficiency may be associated with chronic inflammation or insulin resistance, which can prevent muscle synthesis and induce muscle degradation. Hashemipour et al [30] have reported low PSQI scores in patients with high Homeostasis Model Assessment of Insulin Resistance scores. It has been shown that sleep fragmentation could induce sympathetic overactivity, further leading to insulin resistance [31].

Han et al [32] have reported a bidirectional association between low sleep quality and low grip strength with data from two longitudinal study cohorts and noted that depression partially mediated the association, especially on the impact of grip strength on sleep efficiency. However, in our study, adding GDS-15 scores to the multivariable analysis did not affect the results. The discrepancy may be accounted for by the different patient characteristics and measures used in each study. In Han’s study, the mean age of both cohorts in the study was in the 60s and the test used to assess depression was the Center for Epidemiologic Studies Depression Scale, whereas the patients in our study were in their 80s and the GDS-15 was used.

We observed an association between wearable-estimated sleep efficiency and grip strength in patients with diabetes; however, no significant interaction according to diabetes status was found. Our results did not support the hypothesis that lower sleep efficiency might accelerate insulin resistance, which leads to reduced muscle strength through the direct effect of deceased insulin action and hyperglycemia due to low physical activity. While it has been shown that diabetic peripheral neuropathy (DPN) is associated with both low muscle strength [33] and low sleep quality as evaluated by the PSQI [34], as we did not evaluate DPN, further studies are necessary to clarify whether DPN could affect sleep efficiency and muscle strength in patients with diabetes. Because the association between sleep efficiency and low grip strength remained marginally significant after adjustment for the use of GLP-1 receptor agonists or SGLT2 inhibitors, which may affect muscle mass, these drugs are unlikely to have substantially influenced the results. Obstructive SAS could affect not only sleep efficiency but also muscle strength [35]. However, in our study, sensitivity analyses excluding patients with SAS did not affect the results.

Notably, an association with wearable-estimated sleep efficiency was found only for grip strength and not for physical activity. A possible explanation for this, as approximately half of the participants included in this study had diabetes, could be that in older patients with diabetes, other confounders such as DPN may strongly affect physical function. Indeed, we found no reports showing an association between sleep efficiency and physical function in patients with diabetes.

The strength of this study is that it is the first to use easy-to wear wearable devices with a substantial number of participants and have a long duration of up to 3 months in older outpatients with predominantly cardiometabolic chronic diseases to clarify the association between health lifelogs on devices and physical performance. The most significant advantage of the Silmee device compared to actigraphs is its relatively low cost. It is easily available in retail stores. Therefore, it can be used to monitor sleep indices in real-life settings in numerous outpatients with cardiometabolic diseases.

Although it has been reported that sleep efficiency decreases with age [36], most previous studies were performed among community-dwelling older adults, not hospital outpatients, as in our study. The measured values were objective and more accurate than those obtained from subjective questionnaires. Moreover, step count, which was used as a covariate, was objectively measured using wearable devices. If the data cleaning and processing steps are further refined, simplified, and automated, this has the potential to be a powerful tool both for sleep hygiene education and contributing to muscle health in this population.

### Limitations

However, this study has some limitations. First, due to its cross-sectional design, whether there is a causal relationship between wearable-estimated sleep efficiency and grip strength remains unclear. Further longitudinal studies should be conducted to clarify the influence of wearable-estimated sleep efficiency on physical performance and vice versa. Second, as discussed above, the limited validity of the Silmee device may have influenced the results, although additional analyses incorporating rest-activity rhythm indices supported the robustness of the findings. In addition, because sleep was defined as the period between going to bed after 18:00 and waking before 9:00, irregular sleep patterns could not be evaluated.

Third, as we only measured sleep indices with a wearable device and without the PSQI, a comparison between objective and subjective measures could not be performed in this study. The median sleep length was shorter and the wearable-estimated sleep efficiency was lower than that in previous reports [11,21]. One reason for this could be the differences in patient backgrounds. The participants in our study were older outpatients, most of whom had multiple diseases. Older patients with multimorbidity have lower sleep efficiency [37]. Among the comorbidities, depression was the most influential. We recently reported that depressive symptoms were present in 43.6% of patients with cardiometabolic diseases [38]. Moreover, Zhou et al [39] reported that sleep efficiency as measured using polysomnography in older patients with depression was approximately 60%. Nonetheless, the possibility that Silmee detects sleep times that are shorter than the actual values cannot be excluded.

Fourth, this study was conducted at a single institution; therefore, generalization of the results requires caution. Fifth, in this study, device data were accessed via smartphones by almost all participants who understood how to use the apps after short instructions on usage. This could represent a selection bias. Further, whether the evaluation of health lifelogs with these devices is applicable to all older outpatients remains unclear and requires further investigation.

Finally, several factors that were not evaluated in this study, including pain, dietary and nutritional intake, inflammatory status, undiagnosed SAS, and DPN, may have influenced wearable-estimated sleep efficiency and muscle strength. Because limited or no data on these variables were available, their potential effects could not be fully assessed. Nevertheless, our findings suggest that this inexpensive and easy-to-wear device provides useful information for estimating muscle strength in older patients with cardiometabolic diseases.

### Conclusions

Low sleep efficiency evaluated using wrist-worn wearable devices was associated with low grip strength in older outpatients. This association was observed in patients with diabetes; however, no significant interaction according to diabetes status was found, and there was no evidence that the association differed between patients with and without diabetes. Further longitudinal studies involving larger populations are needed to clarify the long-term association between low wearable-estimated sleep efficiency and muscle health in patients with cardiometabolic diseases.

## Supplementary material

10.2196/95303 Multimedia Appendix 1 Distribution of the valid night count.

10.2196/95303 Multimedia Appendix 2 Supplementary Tables 1-10.

10.2196/95303 Multimedia Appendix 3 Restricted cubic spline models between wearable-estimated sleep efficiency and low grip strength.

## Abbreviations

AWGS: Asian Working Group for Sarcopenia
DPN: diabetic peripheral neuropathy
EEG: electroencephalography
eGFR: estimated glomerular filtration rate
GDS: Geriatric Depression Scale
GLP-1: glucagon-like peptide-1
IS: interdaily stability
IV: intradaily variability
J-CHS: the Japanese version of Cardiovascular Health Study
KCL: Kihon Checklist
MMSE: Mini-Mental State Examination
OR: odds ratio
PSQI: Pittsburgh Sleep Quality Index
RA: relative amplitude
RCS: restricted cubic spline
SAS: sleep apnea syndrome
SGLT2: sodium-glucose cotransporter 2
SMI: skeletal muscle index
SWING-Japan: Smart Watch Innovation for Geriatrics and Gerontology
