# Supplementary material for: Association Between Wrist-Worn Device-Estimated Sleep Efficiency and Hand Grip Strength in Older Outpatients With Cardiometabolic Diseases: Cross-Sectional Study
Source: JMIR Form Res. 2026 Oct 9;10:e95303. doi: 10.2196/95303 (PMC13652524; doi:10.2196/95303)
Supplement: Supplementary file 1 [file formative-v10-e95303-s002.docx]

**Supplementary Table 1. Binominal logistic regression analysis for the association between wearable-estimated sleep efficiency and low grip strength stratified by diabetes**

|  | **Diabetes (-)** | | **Diabetes (+)** | |
| --- | --- | --- | --- | --- |
|  | **OR (95%CI)** | ***P*** | **OR (95%CI)** | ***P*** |
| WESE * | 1.04(0.97-1.11) | .29 | **1.05(1.002-1.093)** | **.039**** |
| Age | **1.16(1.01-1.33)** | **.039** | 1.07(0.97-1.18) | .18 |
| Sex (Men) | 0.64(0.11-3.78) | .62 | 0.87(0.29-2.56) | .79 |
| BMI | 0.90(0.73-1.12) | .35 | 0.96(0.80-1.14) | .61 |
| HbA1c | 0.35(0.10-1.22) | .10 | 0.86(0.41-1.81) | .69 |
| MMSE | 0.89(0.50-1.57) | .68 | 1.18(0.82-1.69) | .37 |
| Daily Steps (per 1000 steps) | 1.06(0.76-1.47) | .74 | 0.89(0.70-1.14) | .36 |
| GDS-15 | 0.92(0.66-1.28) | .63 | 0.909(0.729-1.134) | .40 |

WESE: wearable-estimated sleep efficiency, BMI: body mass index, MMSE: Mini-Mental State Examination, GDS: Geriatric Depression Scale

*1-percentage-point decrease

** *P* for interaction =.458.

**Supplementary Table 2. Wearable-estimated sleep efficiency (%) between patients with and without Low Grip Strength (Limited individuals with≥14 nights data)**

|  | Low grip (-) | Low grip (+) | ***P*** |
| --- | --- | --- | --- |
| Total | **63.3[56.3-79.4]** | **57.5[52.0-62.8]** | **.001** |
| Diabetes (-) | 60.7[56.5-76.7] | 58.3[54.7-62.7] | .181 |
| Diabetes (+) | **66.9[55.6-82.3]** | **55.4[48.3-63.9]** | **.003** |

**Supplementary Table 3. Binominal logistic regression analysis for the association between wearable-estimated sleep efficiency and low grip strength (Model3, Limited individuals with≥14 nights data)**

|  | **Total** | | **Diabetes (-)** | | **Diabetes (+)** | |
| --- | --- | --- | --- | --- | --- | --- |
|  | **OR (95%CI)** | ***P*** | **OR (95%CI)** | ***P*** | **OR (95%CI)** | ***P*** |
| WESE * | **1.05(1.02-1.09)** | **.01** | 1.05(0.97-1.13) | .21 | **1.06(1.01-1.11)** | **.02** |
| Age | 1.07(0.995-1.159) | .07 | 1.14(0.99-1.30) | .07 | 1.08(0.97-1.21) | .18 |
| Sex (Men) | 0.81(0.32-2.02) | .65 | 0.49(0.08-3.11) | .45 | 0.93(0.29-2.96) | .90 |
| BMI | 0.99(0.87-1.12) | .82 | 0.93(0.75-1.14) | .48 | 0.98(0.81-1.19) | .87 |
| HbA1c | 0.92(0.55-1.55) | .76 | 0.39(0.11-1.36) | .14 | 1.16(0.51-2.66) | .73 |
| MMSE | 0.98(0.73-1.32) | .90 | 0.80(0.43-1.49) | .48 | 1.13(0.77-1.64) | .54 |
| Daily Steps (per 1000 steps) | 0.92(0.75-1.12) | .42 | 1.03(0.74-1.44) | .85 | 0.88(0.67-1.16) | .37 |
| GDS-15 | 0.96(0.82-1.14) | .67 | 0.91(0.65-1.26) | .55 | 0.91(0.73-1.14) | .42 |

WESE: wearable-estimated sleep efficiency, BMI: body mass index, MMSE: Mini-Mental State Examination, GDS: Geriatric Depression Scale

*1-percentage-point decrease

**Supplementary Table 4. Wearable-estimated sleep efficiency (%) between patients with and without Low Grip Strength (Limited individuals with≥30 nights data)**

|  | Low grip (-) | Low grip (+) | ***P*** |
| --- | --- | --- | --- |
| Total | **61.4[55.4-79.0]** | **56.6[52.0-62.3]** | **.005** |
| Diabetes (-) | 60.2[56.5-76.7] | 58.3[54.7-62.7] | .221 |
| Diabetes (+) | **61.5[53.8-82.0]** | **53.8[48.3-61.1]** | **.010** |

**Supplementary Table 5. Binominal logistic regression analysis for the association between wearable-estimated sleep efficiency and low grip strength (Model3, Limited individuals with≥30 nights data)**

|  | **Total** | | **Diabetes (-)** | | **Diabetes (+)** | |
| --- | --- | --- | --- | --- | --- | --- |
|  | **OR (95%CI)** | ***P*** | **OR (95%CI)** | ***P*** | **OR (95%CI)** | ***P*** |
| WESE * | **1.06(1.01-1.10)** | **0.01** | 1.05(0.97-1.12) | .23 | **1.07(1.01-1.14)** | **.02** |
| Age | **1.09(1.002-1.176)** | **0.045** | 1.13(0.99-1.30) | .07 | 1.11(0.97-1.26) | .13 |
| Sex (Men) | 0.81(0.29-2.27) | 0.69 | 0.61(0.09-4.09) | .61 | 0.79(0.20-3.12) | .74 |
| BMI | 0.96(0.84-1.11) | 0.59 | 0.92(0.75-1.14) | .45 | 0.96(0.75-1.21) | .70 |
| HbA1c | 0.91(0.52-1.58) | 0.74 | 0.42(0.12-1.41) | .16 | 1.39(0.52-3.78) | .51 |
| MMSE | 0.97(0.71-1.34) | 0.87 | 0.85(0.45-1.60) | .61 | 1.14(0.74-1.74) | .54 |
| Daily Steps (per 1000 steps) | 0.94(0.76-1.16) | 0.54 | 1.04(0.75-1.44) | .82 | 0.87(0.63-1.20) | .40 |
| GDS-15 | 0.92(0.75-1.11) | 0.37 | 0.95(0.67-1.34) | .76 | 0.78(0.58-1.05) | .10 |

WESE: wearable-estimated sleep efficiency, BMI: body mass index, MMSE: Mini-Mental State Examination, GDS: Geriatric Depression Scale

*1-percentage-point decrease

**Supplementary Table 6. Wearable-estimated sleep efficiency (%) between patients with and without Low Grip Strength (excluding users of sleep medications or antipsychotics)**

|  | Low grip (-) | Low grip (+) | ***P*** |
| --- | --- | --- | --- |
| Total | **62.0[55.8-78.5]** | **58.8[52.7-64.1]** | **.027** |
| Diabetes (-) | 60.1[55.3-76.5] | 58.5[53.7-62.5] | .295 |
| Diabetes (+) | **68.4[55.9-81.0]** | **59.1[50.3-68.6]** | **.030** |

**Supplementary Table 7. Binominal logistic regression analysis for the association between wearable-estimated sleep efficiency and low grip strength (Model3, excluding users of sleep medications or antipsychotics)**

|  | **Total** | | **Diabetes (-)** | | **Diabetes (+)** | |
| --- | --- | --- | --- | --- | --- | --- |
|  | **OR (95%CI)** | ***P*** | **OR (95%CI)** | ***P*** | **OR (95%CI)** | ***P*** |
| WESE * | **1.04(1.002-1.08)** | **.04** | 1.06(0.98-1.16) | .15 | 1.04(0.99-1.09) | .11 |
| Age | **1.10(1.01-1.19)** | **.03** | **1.24(1.04-1.48)** | **.02** | 1.09(0.98-1.21) | .13 |
| Sex (Men) | 1.08(0.42-2.81) | .87 | 1.86(0.19-18.5) | .59 | 0.90(0.27-2.98) | .87 |
| BMI | 0.94(0.82-1.08) | .40 | 0.86(0.65-1.13) | .28 | 0.93(0.76-1.13) | .46 |
| HbA1c | 0.66(0.36-1.20) | .17 | 0.14(0.02-1.23) | .08 | 0.60(0.24-1.49) | .27 |
| MMSE | 1.05(0.77-1.43) | .87 | 0.84(0.43-1.67) | .62 | 1.20(0.79-1.81) | .39 |
| Daily Steps (per 1000 steps) | 0.97(0.79-1.19) | .77 | 1.20(0.82-1.74) | .35 | 0.91(0.69-1.18) | .47 |
| GDS-15 | 1.03(0.85-1.25) | .76 | 1.05(0.72-1.53) | .81 | 0.98(0.74-1.30) | .90 |

WESE: wearable-estimated sleep efficiency, BMI: body mass index, MMSE: Mini-Mental State Examination, GDS: Geriatric Depression Scale

*1-percentage-point decrease

**Supplementary Table 8. Wearable-estimated sleep efficiency (%) between patients with and without Low Grip Strength (excluding sleep apnea syndrome)**

|  | Low grip (-) | Low grip (+) | ***P*** |
| --- | --- | --- | --- |
| Total | **62.1[56.3-79.1]** | **58.1[52.9-64.0]** | **0.008** |
| Diabetes (-) | 60.7[56.5-76.7] | 58.3[54.7-62.7] | 0.195 |
| Diabetes (+) | **65.4[55.6-82.5]** | **57.8[48.9-66.2]** | **0.013** |

**Supplementary Table 9. Binominal logistic regression analysis for the association between wearable-estimated sleep efficiency and low grip strength (Model3, excluding sleep apnea syndrome)**

|  | **Total** | | **nonDM** | | **DM** | |
| --- | --- | --- | --- | --- | --- | --- |
|  | **OR (95%CI)** | ***P*** | **OR (95%CI)** | ***P*** | **OR (95%CI)** | ***P*** |
| WESE * | **1.05(1.01-1.09)** | **.01** | 1.04(0.97-1.11) | .28 | **1.05(1.005-1.106)** | **.03** |
| Age | **1.08(1.004-1.166)** | **.04** | **1.15(1.002-1.319)** | **.047** | 1.08(0.98-1.20) | .12 |
| Sex (Men) | 0.96(0.39-2.38) | .93 | 0.62(0.10-3.72) | .60 | 1.05(0.34-3.31) | .93 |
| BMI | 0.94(0.82-1.09) | .42 | 0.93(0.74-1.15) | .49 | 0.92(0.74-1.13) | .42 |
| HbA1c | 0.79(0.46-1.35) | .39 | 0.34(0.10-1.21) | .10 | 0.93(0.43-2.03) | .85 |
| MMSE | 1.03(0.77-1.38) | .83 | 0.89(0.50-1.57) | .68 | 1.20(0.82-1.74) | .34 |
| Daily Steps (per 1000 steps) | 0.96(0.79-1.17) | .69 | 1.05(0.76-1.46) | .76 | 0.91(0.71-1.18) | .48 |
| GDS-15 | 0.96(0.81-1.15) | .66 | 0.90(0.65-1.26) | .55 | 0.92(0.73-1.16) | .48 |

WESE: wearable-estimated sleep efficiency, BMI: body mass index, MMSE: Mini-Mental State Examination, GDS: Geriatric Depression Scale

*1-percentage-point decrease

**Supplementary Table 10. Binominal logistic regression analysis for the association between wearable-estimated sleep efficiency and low grip strength (Model3 plus RA_7)**

|  | **Total** | | **nonDM** | | **DM** | |
| --- | --- | --- | --- | --- | --- | --- |
|  | **OR (95%CI)** | ***P*** | **OR (95%CI)** | ***P*** | **OR (95%CI)** | ***P*** |
| WESE * | **1.04(1.002-1.073)** | **.04** | 1.03(0.97-1.10) | .36 | 1.04(0.997-1.091) | .07 |
| Age | 1.07(0.995-1.148) | .07 | 1.16(0.995-1.339) | .06 | 1.07(0.97-1.18) | .18 |
| Sex (Men) | 0.87(0.36-2.10) | .76 | 0.57(0.09-3.46) | .54 | 0.86(0.27-2.67) | .80 |
| BMI | 0.95(0.84-1.08) | .44 | 0.90(0.73-1.11) | .33 | 0.95(0.79-1.13) | .54 |
| HbA1c | 0.80(0.48-1.35) | .41 | 0.35(0.10-1.19) | .09 | 0.85(0.39-1.88) | .69 |
| MMSE | 1.05(0.79-1.39) | .75 | 0.91(0.51-1.62) | .75 | 1.19(0.83-1.72) | .35 |
| Daily Steps (per 1000 steps) | 0.94(0.77-1.13) | .49 | 1.12(0.77-1.64) | .55 | 0.89(0.70-1.13) | .34 |
| GDS-15 | 0.96(0.81-1.13) | .60 | 0.90(0.66-1.28) | .62 | 0.91(0.72-1.14) | .39 |
| RA_7 | 0.38(0.02-8.38) | .54 | 0.21(0.00-140.2) | .64 | 0.53(0.01-27.3) | .75 |

WESE: wearable-estimated sleep efficiency, BMI: body mass index, MMSE: Mini-Mental State Examination, GDS: Geriatric Depression Scale, RA_7: relative amplitude over consecutive seven days

*1-percentage-point decrease
